# Outer Limits of Biotechnologies: A Jewish Perspective

**DOI:** 10.5041/RMMJ.10328

**Published:** 2018-01-29

**Authors:** John D. Loike, Alan Kadish

**Affiliations:** 1 Department of Pathology, Columbia University, New York, NY, USA; 2 Touro College, New York, NY, USA; 3 President, Touro College and University System, New York, NY, USA

**Keywords:** Bioethics, gene editing, medical ethics

## Abstract

A great deal of biomedical research focuses on new biotechnologies such as gene editing, stem cell biology, and reproductive medicine, which have created a scientific revolution. While the potential medical benefits of this research may be far-reaching, ethical issues related to non-medical applications of these technologies are demanding. We analyze, from a Jewish legal perspective, some of the ethical conundrums that society faces in pushing the outer limits in researching these new biotechnologies.

## INTRODUCTION

Society is in the midst of a technological revolution, especially in reproductive medicine and molecular genetics, areas that hold the promise of improving human health in unimaginable ways. In 2017, for example, scientists have applied gene-editing (clustered regularly interspaced short palindromic repeats, referred to as CRISPR) technology to correct the genetic cause of a fetal heart disease, hypertrophic cardiomyopathy, and a common blood disease, β-thalassemia.^1^,^2^ The use of this technology *in utero* can enable babies to be born without these serious and sometimes fatal diseases. Another example of potentially life-saving advances in biotechnology and organ transplantation is using human stem cells to create chimeric pigs that develop human organs.^3^–^5^

These scientific achievements are both exciting and ethically challenging.^6^,^7^ The excitement is the hope that gene-editing technology can be useful in correcting some of the over 10,000 mutations that can cause human disease. Creating chimeric human-pigs offers a unique opportunity for people whose own livers, kidneys, or hearts are irreversibly failing, to safely receive organs donated from animals.

The ethical concerns regarding these biotechnologies are many. Will society limit their use to curing disease, or will also people begin to use technology for non-medical purposes? Creating “designer babies” such as those with particular facial structures or enhanced intelligence is one such concern that has been problematic since the days of the original 1967 Star Trek episode, “Space Seed” (which inspired the film *Star Trek II: The Wrath of Khan*). Another group of science fiction writers who penned *Planet of the Apes* note that there is also a fear of creating animals with reconstituted human brains or human speech and of placing people at risk of unknown side effects. Moreover, some people believe that such technologies are unethical because they enable human beings to “play God” by tampering with the holy genetic grail or interfering with the normal evolution of species.

## BALANCING SCIENTIFIC INNOVATION WITH ETHICAL CONCERNS

How should society balance the innate human desire to innovate new technologies with the fear that their applications may violate ethical norms? While these debates are ongoing, current United States government guidelines ban the use of federal funds to modify embryos using technologies such as CRISPR. If science has the capacity to save lives through such technology, are we ethically justified in not moving this forward?

When viewed through the Jewish lens of the Bible (written law) and the Talmud (oral law), there is precedent for legal and moral deliberations of this kind. Jewish philosophy and law have debated similar issues over the past two thousand years and offer meaningful guidelines that address the question of balancing scientific discovery with the fear of unethical applications. Great rabbinical thinkers from Nachmanides (thirteenth century) to Rabbi Joseph Soloveitchik (twentieth century) stated that God created an incomplete world in which human beings must utilize their creative capacities to complete the creation process. In other words, God directed human beings to partner with Him to finish the creation process.^8^–^10^

## PLAYING GOD—MORAL IMPERATIVE?

The lessons from these discussions relate most profoundly to current times. We propose that a Divine directive is for human society to embrace science by actively supporting the research of natural law and applying it wisely. This Divine directive represents a universal directive for all humanity. Clearly, the Bible and Talmud understood that human inhabitation of this world requires scientific research that encompasses biology, chemistry, and physics to enable human beings to live in an environmentally, biologically, and medically healthy society. Thus, the concept of human beings “playing God” can be viewed as an ethical principle not an immoral activity.

Judaism recognizes that scientific research begins by understanding basic principles underlying biological and cellular processes through observation of the wide variety of biological life. Biological precedent is an important guideline in both scientific discovery and applications. In fact, scientists incorporate this philosophical approach today. Gene editing is one example of a scientific discovery that originated from basic research in virology that showed how a simple bacterium can protect itself from an invading virus.^11^

Jewish law also presents a profound directive from the classical moral objective that one is obligated to “refrain from doing bad and do good.”^12^,^13^ Judaism believes that the ideal objective is to *transform the evil* into goodness. The Talmud states that the answers to curing human diseases lie within the laws of nature because God created the cures for all diseases even before He created disease pathology.^14^ There is no better example of this concept than what we are currently witnessing in the excitement surrounding immunotherapy and cancer.^15^ Understanding why our immune system fails to fight tumors has enabled scientists to re-educate our T cells to fight and destroy lethal cancers.

## A PATH FOR ETHICAL ANALYSIS

The challenge is how to apply scientific research in practice in a morally acceptable manner. The Supreme Court of Jewish Law would always address religious legal questions at two levels in a manner similar to how the US supreme court deals with difficult cases.^16^,^17^ The first level was a general, theoretical legal analysis of the history of law and how the populace practiced the parameters of a law. The second level was a practical and particular approach, examining each situation according to the individual circumstances and developing the response according to the specific details and characteristics of that situation. Indeed, Jewish law places a much greater emphasis on the latter principle—each case must be evaluated individually on its merits and details.

We believe there is much merit in this type of analysis regarding the new biotechnologies being developed. First, we must ethically analyze the various procedures underlying the biotechnology. Does it involve harming animals? Does it violate basic principles of bioethics? Then, we should address these principles as they apply to a particular situation and recognize that all technologies, while morally neutral, have the potential to do both good and/or to do harm.

Translational biotechnology, in order to develop new therapies for disease, is the ultimate objective of science and is part of the Divine directive for human beings to partner with God. However, society must be careful of the outer limits of technology that may be unethical. Do the ethical outer limits of technology include non-medical procedures such as sex-selection, cosmetic changes (hair or eye color, height), or tampering with biological species to create new species, such as animals with human neurons, or monkeys with human genes that regulate human speech?

The general rule in Judaism is that gene editing for non-medical applications is ethically wrong and should not be routinely acceptable. In the case of gene-editing a human embryo, we believe it is moral and ethical to genetically edit not only an embryo carrying lethal genes (e.g. Tay–Sachs) but also in cases where the child would be born and burdened with serious health issues (e.g. cystic fibrosis).

The Torah states that its laws are created for people to live by, and so we should support medical and technological advances that promote the saving of lives. We should advocate that society commit significant government and private funding to basic research. This also includes supporting research for creative procedures that protect the weaker segments of the population in order to provide fair allocation of treatments. We advocate continuing to push the limits of scientific research, but at the same time, we must not allow the unethical, non-medical applications. In the realm of new biotechnology, the goal of partnering with God to save lives should be paramount. It is not what you can do but what you should do.

## Abbreviations

CRISPR: clustered regularly interspaced short palindromic repeats
